# Central Role of Ubiquitination in Genome Maintenance: DNA Replication and Damage Repair

**DOI:** 10.5402/2012/146748

**Published:** 2012-02-08

**Authors:** Soma Ghosh, Tapas Saha

**Affiliations:** ^1^Department of Oncology, Sidney Kimmel Comprehensive Cancer Center, Johns Hopkins School of Medicine, Johns Hopkins University, Baltimore, MD 21231, USA; ^2^Department of Oncology, Lombardi Comprehensive Cancer Center, Georgetown University Medical Center, Washington, DC 20057, USA

## Abstract

Faithful transmission of genetic information through generations ensures genomic stability and integrity. However, genetic alterations occur every now and then during the course of genome duplication. In order to repair these genetic defects and lesions, nature has devised several repair pathways which function promptly to prevent the cell from accumulating permanent mutations. These repair mechanisms seem to be significantly impacted by posttranslational modifications of proteins like phosphorylation and ubiquitination. Protein ubiquitination is emerging as a critical regulatory mechanism of DNA damage response. Non-proteolytic, proteasome-independent functions of ubiquitin involving monoubiquitination and polyubiquitination of DNA repair proteins contribute significantly to the signaling of DNA repair pathways. In this paper, we will particularly highlight the work on ubiquitin-mediated signaling in the repair processes involving the Fanconi anemia pathway, translesional synthesis, nucleotide excision repair, and repair of double-strand breaks. We will also discuss the role of ubiquitin ligases in regulating checkpoint mechanisms, the role of deubiquitinating enzymes, and the growing possibilities of therapeutic intervention in this ubiquitin-conjugation system.

## 1. Introduction

DNA damage response pathways have been evolved to maintain the genomic integrity of organisms as well as to counter serious assaults on genomic stability. Errors made by the DNA replication machinery can cause genomic instability and contribute significantly to the onset of cancer [1]. Several pathological diseases are linked with aberrant DNA replication through several DNA mutations and chromosome rearrangements [2]. Moreover, faithful transfer of genetic information during replication can be impaired by several environmental and cellular factors like replication stress, reactive oxygen species (ROS), reactive nitrogen species (RNS), and exposure to UV or ionizing radiation. Highly transcribed DNA sequences, several secondary DNA structures, and modified/damaged DNA stall replication forks. To prevent the deleterious effects of DNA damage, several checkpoint responses are activated following the damage. The checkpoint response can repair the damaged DNA prior to the next round of cell division or it can signal the cell to undergo apoptosis. DNA can be damaged by the introduction of single-strand breaks (SSBs) and/or double-strand breaks (DSBs) and/or formation of DNA adducts (crosslinking of individual purine or pyrimidine bases). DNA damage sensors and repair proteins act promptly to remove these lesions in a timely manner so that the genome is protected from permanent mutations [3]. Eukaryotic cells have developed several repair pathways in order to maintain genomic stability and integrity. The major repair pathways are mismatch repair (MMR), nucleotide excision repair (NER), base excision repair (BER), homologous recombination (HR), nonhomologous end joining (NHEJ), and translesion synthesis (TLS).

Posttranslational modifications of several key regulatory proteins help in maintaining genomic integrity. The scope and role of protein phosphorylation is well established in the DNA repair pathways, but the role of protein ubiquitination has recently been reported as a key regulatory mechanism that influences almost all aspects of the DNA repair pathways. Ubiquitin, a highly conserved, 76-aminoacid protein is commonly used by cells for proteasome-mediated protein degradation. However, its proteasome-independent functions help in the regulation of DNA repair mechanisms. Ubiquitination regulates the activities of the ATP-dependent, ubiquitin-activating enzyme (E1), ubiquitin-conjugating enzyme (E2), and ubiquitin ligase (E3) [4, 5]. However, this can be reversed by a family of enzymes known as the deubiquitinating enzymes (DUBs) [6, 7].

Monoubiquitination is an addition of a single ubiquitin molecule to the substrate and is involved in a wide variety of cellular functions. It ranges from control of endocytosis, and intravesicular transport [8] to transcriptional regulation (ubiquitination of histone H2A on lysine 119 is required for polycomb group gene silencing and X-chromosome inactivation) [9, and references therein], DNA replication, and repair [10]. The activity of the mammalian origin recognition complex (ORC) is regulated by cell-cycle-dependent changes in its Orc1 subunit. Modification of Orc1 in the form of monoubiquitination and phosphorylation during S and G2-M phases is essential for mammalian development. In the absence of these modifications, p53-independent apoptosis occurs in the cells leading to genomic instability [11–14]. Polyubiquitination or the ability of ubiquitin molecules to form a polymeric chain adds another layer of complexity to ubiquitin-mediated signaling [15]. All the seven lysine residues of ubiquitin can act as initiators of ubiquitin polymeric chains. In addition, the amino terminus of ubiquitin also acts as an acceptor for the formation of polymeric ubiquitin chains. As the lysine residues are distributed over the surface of the ubiquitin molecule, chains of different linkage produce different geometries and thus result in generation of structurally distinct signals with unique consequences for the modified substrate [16].

In this paper, we will highlight the role of ubiquitination in major repair pathways: the repair mechanism after DSBs is mediated by polyubiquitination, the repair of DNA adducts by nucleotide excision repair pathway, and the Fanconi anemia (FA) pathway. Repair of interstrand crosslinks (ICLs) during DNA replication is mediated by monoubiquitination, and DNA damage tolerance for replicative lesion bypasses by both monoubiquitination and polyubiquitination. We will also discuss the role of ubiquitin ligases in the regulation of checkpoint functions, functions of deubiquitinating enzymes, and finally the possibilities of the ubiquitin signaling mechanism as a therapeutic target for cancer.

## 2. DNA Damage during DNA Replication

Many lesions are known to interfere with DNA replication. During S-phase, DNA damage is sensed due to inhibitory effects on DNA polymerase. This results in replication fork stalling, and activation of the checkpoint kinase ataxia telangiectasia and Rad3-related (ATR) [17]. Once a replication fork stalls, repair pathways are activated to allow re-initiation of replication. Collapse of replication forks also triggers a checkpoint response that results in cell cycle arrest, DNA repair, or cell death through apoptosis. The collapse of forks can be avoided by bypassing the lesions in a process known as translesion synthesis or TLS [18, 19]. A growing class of DNA polymerases designated alphabetically *ζ* to *κ* have recently been reported to be involved in repairing damage-induced replication stress [20, 21]. These special polymerases, also known as translesion polymerases, have flexible base-pairing properties and hence permit translesion synthesis by temporarily taking over from the blocked replicative DNA polymerase-*δ*/*ε* and pol*α*. Since the translesion polymerases have low fidelity, they are responsible for introducing several point mutations in the genome, leading to permanent damage and onset of carcinogenesis. In yeast (*S. cerevisiae*), a second pathway exists that ensures error-free bypass of DNA damage or lesions [22]. This mechanism involves reinitiation of replication downstream of the lesion with the resultant gap filled in by recombination replication using the newly synthesized complementary strand. Yeast protein complexes, like Ubc13/Mms2, are involved in this process, and are conserved all the way to mammals. Therefore, this pathway in humans needs to be explored.

Interstrand DNA crosslinks (ICLs) are extremely toxic DNA lesions and are particularly deleterious for the cell as they prevent the separation of DNA strands required for both replication and transcription. The repair of ICL is mediated by a group of factors that belong to the FA pathway. During DNA replication, monoubiquitination of two components of the FA pathway, FANCD2 and FANCI, triggers or activates ICL repair.

Double-strand breaks (DSBs) in the double helix during DNA replication are particularly hazardous to cells because they can obstruct replication fork progression and result in genome rearrangements. DSBs are considered the most toxic of all DNA lesions. DSBs can be repaired by homologous recombination and nonhomologous end joining. The damage response to a DSB involves complex ubiquitin-mediated signaling events that lead to the recruitment of modified proteins at the site of damage as detailed later in this paper. The ubiquitin system mediates the DNA damage response to all the above forms of replicative damage in the cell in order to prevent genomic instability and onset of cancer. Another common form of DNA damage that interferes with the replication fork progression is the chemical modification (adducts) of DNA bases that occur due to interaction with chemically reactive drugs or exposure to UV or ionizing radiation. Small adducts can be identified and removed by base excision repair (BER) pathway, whereas the bulky ones are removed by nucleotide excision repair (NER) pathway [10]. BER pathway is highly conserved among species. It is a high fidelity DNA repair mechanism that occurs during repair of oxidative DNA lesion in the genome. Recently it was reported that BER is regulated by breast cancer susceptibility gene 1 (BRCA1), which is a tumor suppressor for the hormone-responsive cancers like breast, prostate, and ovarian cancer. BRCA1 upregulates the key enzymes of the BER pathway such as OGG1, Nth1, and Ref1 via the Oct1 transcription factor to stimulate the process during oxidative stress [23, 24].

## 3. Ubiquitination of the Checkpoint Proteins that Control Genome Surveillance

The cellular machinery responsible for DNA damage surveillance and subsequent repair has the unique property of sensing modification of the DNA and then arresting the cells at specific cell cycle checkpoints. This allows repair of damaged DNA in order to avoid their transformation into permanent damage or mutations [25]. DNA damage checkpoints occur at the G1/S and G2/M boundaries as well as to an intra-S checkpoint during the cell cycle. ATM and ATR are the two essential kinases that control the checkpoint activation. Structural modification of the chromatin and the DNA double-strand breaks are the substrates of ATM [26, 27], while ATR is primarily recruited in the stalled replication forks [28]. A class of checkpoint mediator proteins including BRCA1, MDC1, and 53BP1 has been identified and will be discussed in this paper. The utmost posttranslational modifications implicated in the regulation of checkpoint activation are phosphorylation and ubiquitination [29, 30]. The DNA damage checkpoint activation ensures arresting or slowing down of the cell cycle, so that the cell has adequate time to either repair the lesions before entering the next cell division cycle or undergo apoptosis. In mammalian cells, the ATM-CHK2 and ATR-CHK1 kinase-pathways play key roles in signaling checkpoint arrest [31]. The cyclin-dependent kinases (CDKs) are the prime targets of the checkpoint pathways, but they are not targeted by the checkpoint kinases ATM or CHK1. The checkpoint apparatus targets the CDK regulators like cyclins, CDK inhibitors, or CDC25 family of dual-specificity phosphatases, depending upon the stage of the cell cycle in which the DNA damage has occurred.

Two types of ubiquitin ligases the SCF (Skp1/Cul1/F-box) and the APC/C play central roles in cell cycle regulation. The phosphatase CDC25A is degraded by the ubiquitin-proteasome machinery [32, 33] in response to DNA damage, resulting in CDK inhibition and cell cycle arrest. CHK1/CHK2-mediated phosphorylation of CDC25A results in recognition of phosphorylated CDC25A by SCF^ßTrCP^, leading to the degradation of CDC25A [34, 35]. SCF^ßTrCP^ also regulates checkpoint recovery: the ubiquitin-dependent degradation of CLASPIN (a DNA-binding protein required for the ATR mediated activation of Chk1 in response to DNA replication stress) in G2 allows efficient termination of DNA replication checkpoint which is necessary for progression of the cell into mitosis [36–39].

The E3 ubiquitin ligase APC/C which is active during M and G1 phases of the cell cycle, helps in the formation of polyubiquitin chains on substrates for subsequent degradation [40, 41]. APC/C either associates with subunit CDC20 to form APC/C^CDC20^ that mediates its proteasomal degradation during G1 or associates with subunit CDH1 to form APC/C^CDH1^, which functions during the G2/M phase of the cell cycle. During G1 phase of the cell cycle, APC^CDH1^ takes part in a p53-independent checkpoint response that targets the degradation of cyclin D1 [40]. During G2/M checkpoint, DNA damage triggers the activation of APC^CDH1^, which is mediated by an ATM-independent repair mechanism. Upon checkpoint activation, phosphatase CDC14B is translocated from the nucleus to the nucleoplasm, resulting in dephosphorylation of CDH1. The dephosphorylated CDH1 thereafter activates the APC/C.

## 4. Role of Monoubiquitination in Fork Block Lesions during DNA Replication

### 4.1. Regulation of Fanconi Anaemia Pathway

Interstrand crosslinks (ICLs) are known to prevent strand separation during both transcription and replication, and its repair is collectively mediated by several DNA repair proteins known as the Fanconi anaemia (FA) pathway. In addition to removal of the lesion, the FA pathway is also responsible for sensing the ICLs and thereafter triggering an appropriate ATR-dependent checkpoint response [42]. Monoubiquitination of two FA components by other members of the pathway leads to the activation of ICL repair during DNA replication.

**Figure 1:**
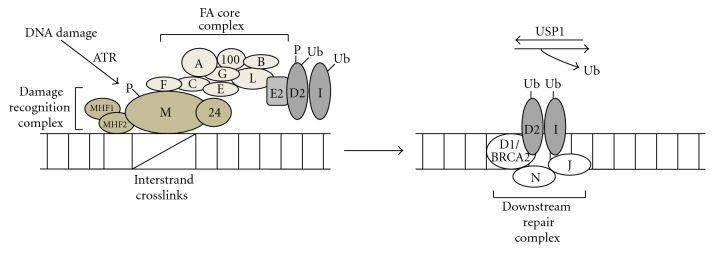
Fanconi's anemia pathway. DNA ICLs are recognized by a recognition complex consisting of FANCM, protein FAAP-24, and histone fold proteins MHF1 and MHF2. This complex then loads the FA core complex comprising of FANC (A, B, C, E, F, G, L, and FAAP-10) subunits and having the E3 ligase activity. FANCL, the catalytic subunit of the complex, interacts with the E2 conjugating enzyme. FANCL binds FANCD2 and FANCI, monoubiquitinates them, and finally recruits them to chromatin. Monoubiquitinated FANCD2 is finally targeted to the repair foci along with BRCA2, FANCJ, FANCN, and other proteins to carry out the repair function.

DNA interstrand cross links are recognized by a protein complex comprising FANCM, FA-associated protein 24 (FAAP-24), and DNA-binding histone fold proteins MHF1 and MHF2 as shown in Figure 1. This recognition complex then recruits the FA core complex (comprising FANCA, B, C, E, F, G, L, and FAAP-100) onto the site of the DNA lesion. FANCL is the catalytic subunit of the core complex and interacts with the E2 conjugating enzyme (UBE2T) to ubiquitinate FANCD2 and FANCI, which are then recruited to the damaged chromatin. Monoubiquitination of the FANCD2 protein takes place at Lysine 561 and is conserved among eukaryotes, suggesting the evolutionary significance of this particular gene. A mutation in Lysine 561 affecting the monoubiquitination of this protein fails to complement the DNA crosslinking activity of FANCD2-deficient cells [43, 44]. Monoubiquitinated FANCD2 appears to colocalize with other components that are recruited to the site of DNA repair including BRCA1, BRCA2, RAD51, PCNA, and REV1 suggesting a functional connection between the FA pathway and the homologous recombination and TLS pathways [45–47]. As a result, the presence of FANCD2 at the DNA damage foci depends upon its ubiquitination at K561, demonstrating the importance of monoubiquitinated form of FANCD2 as a targeting signal to the sites of DNA damage [43]. FANCI (mutated in FA-I patients) also undergoes monoubiquitination and this modification is critical for repair of DNA crosslink damage [48]. The current understanding is that, in the absence of DNA damage, FANCD2 and FANCI are present as free entities. DNA damage triggers the FA pathway thus modifying FANCD2 and FANCI by monoubiquitination and phosphorylation. Special receptors at the sites of DNA damage recognize these modified substrates and target them to the DNA repair foci. The breast and ovarian cancer susceptibility protein BRCA1 also plays a significant role in the FA pathway. A conserved RING domain in BRCA1 forms a heterodimer with the RING domain of another protein BARD1. The BRCA1-BARD1 complex monoubiquitinates FANCD2 *in vitro*. However, the heterodimer does not seem to influence FANCD2 monoubiquitination *in vivo*. The formation of the FANCD2 repair foci is severely impaired by depletion of BRCA1, but the specific role of BRCA1 in FANCD2 monoubiquitination is still unclear. Monoubiquitinated FANCD2 interacts with the downstream repair proteins BRCA2/FANCD1 and possibly FANCJ and FANCN to repair the DNA crosslinks and other lesions by homologous recombination. 

In addition to the monoubiquitination of FANCD2, deubiquitination of FANCD2 by deubiquitination enzyme USP1 is known to be required for ICL repair. An USP1 knockout mouse showed an increased chromatin localization of monoubiquitinated FANCD2 without proper assembly of FANCD2 repair foci and subsequent defects in homologous recombination [49]. Deficiency of USP1 or FANCD2 promotes haematopoietic stem cells defects [50], and siRNA knockdown experiments have shown that USP1 also deubiquitinates FANCI [48], but whether this step is indispensable for ICL repair needs to be ascertained.

### 4.2. Regulation of Translesion Synthesis

The sliding clamp of DNA replication, also known as the proliferative cell nuclear antigen (PCNA), plays a leading role in the regulation of replicative lesion bypass (reviewed in [51]). PCNA in budding yeast is subject to ubiquitination and sumoylation at Lys 164 [52]. Monoubiquitination of PCNA is mediated by the E2 ubiquitin-conjugating enzyme Rad6 and the Rad18 RING finger-containing E3-ubiquitin ligase [52, 53]. Monoubiquitination of PCNA at a stalled replication fork leads to the recruitment of several damage-tolerant DNA polymerases at the site of damage [53–55]. These enzymes use the damaged DNA as a template for translesion synthesis and their actions on different lesions lead to mutagenesis as the translesion polymerases have exceptionally low fidelity. Polyubiquitination of PCNA, mediated by the combined action of Rad6, Rad18, and Rad5 ubiquitin ligases, promotes an alternative error-free pathway of bypassing the damage [56]. Recent studies have shown that monoubiquitinated-PCNA is deubiquitinated by the deubiquitinating enzyme USP1 [10]. siRNA knockdown of USP1 results in increased levels of monoubiquitinated-PCNA both in the presence and absence of DNA damage. Therefore, USP1 seems to regulate the error-prone translesion synthesis activity in the cell in the absence of UV-induced DNA damage.

## 5. Role of Polyubiquitination in DNA Double-Strand Break Signaling and Repair

### 5.1. The RNF8 Pathway

**Figure 2:**
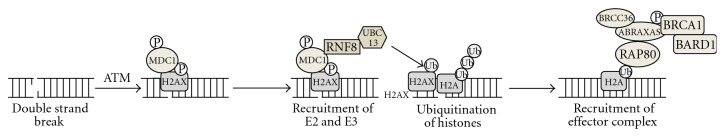
RNF8-mediated ubiquitination at double-strand breaks. Upon DNA damage, checkpoint kinase ATM phosphorylates H2AX which in turn phosphorylates MDC1 and recruits it to the site of DNA damage. MDC1 then recruits RNF8, the E3 ubiquitin ligase, which conjugates with its E2-conjugating enzyme, Ubc13 to ubiquitinate several variant histones. This leads to the loading of the downstream repair proteins including Abraxas-Brca1-Brcc36 through the ubiquitin interaction domain of RAP80. Brcc36 is a deubiquitinating enzyme, which can deubiquitinate the ubiquitin substrates and reverse this RNF8 pathway.

The DNA damage response following a DSB triggers a series of ubiquitination enzymes that lead to checkpoint activation. Signaling at the DSB depends mainly on the multifunctional E3 enzyme (BRCA1). Recruitment of BRCA1 to the site of damage is mediated by a series of ubiquitination events that are initiated by RING finger protein 8 (RNF8) in complex with the E2 ubiquitin-conjugating enzyme 13 (Ubc13) [57–60]. Protein kinases ATM and ATR activate signaling cascades that recruit repair proteins to the sites of DNA damage. The ATM protein kinase is activated and recruited at the site of DSBs through the Mre11-Rad50-Nbs1 or the MRN sensor complex (reviewed in [61]), and then it phosphorylates several proteins such as H2AX [62]. H2AX is one of several genes that codes for histone H2A. Phosphorylated H2AX recruits the ATM substrate, MDC1 [63], and finally leads to the recruitment of the repair machinery at the site of damage. The ring finger protein RNF8, in conjugation with its E2 enzyme Ubc13, ubiquitinylates H2AX and H2A thus leading to recruitment of proper effector complexes like Abraxas-BRCA1-Brcc36 complex at the sites where the repair foci are formed, as shown in Figure 2. BRCA1 is recruited to the DNA damage sites through the ubiquitin-interaction-motif-(UIM-) containing protein Rap 80 and is associated with the BRCA1-binding protein Abraxas [64–67]. Although there are reports of formation of functional complex between BRCA1 and H2AX [68], it is still unclear if polyubiquitination of H2AX and H2A is essential for recruitment of BRCA1 or RAP 80, or if the polyubiquitination of the H2AX and H2A is a consequence of RNF8 recruitment. Depletion of RNF8 leads to a failure in assembling the different components at the sites of damage. Thus, RNF8 may serve as the leading player responsible for orchestrating the events associated with damage at the double-strand break. Also, cells lacking the RNF8-E2 ubiquitin conjugating enzyme, Ubc13, fail to establish a proper DNA damage response [69]. In addition, loss of Ubc13 decreases H2AX monoubiquitination and reduces damage-triggered H2AX polyubiquitination. Finally, once the repair process is completed through the function of the ubiquitin-mediated signaling complex initiated by RNF8, deubiquitinating enzymes (DUBs) remove or edit most of the polyubiquitin chains [70] to disassemble the repair complexes after the double-strand break repair is completed.

### 5.2. Nucleotide Excision Repair (NER) Pathway

The bulky DNA adducts that are introduced by exposure to several chemical compounds or UV radiation are recognized and removed by the NER pathway. These helix-distorting damages include the UV-induced cyclobutane pyrimidine dimers and pyrimidine-pyrimidone (6-4) photoproducts (6-4PP). Defective NER activity gives rise to some rare autosomal-recessive disorders in humans such as xeroderma pigmentosum (XP) and the Cockayne syndrome (CS). In the NER pathway, thirty different proteins function together for detection of the DNA lesion, opening of the helix, incision on the damaged DNA strand, removal of the lesion-containing DNA strand, DNA synthesis for gap-filling, and finally DNA ligation. NER operates in two pathways: the first pathway repairs damages across the entire genome known as global genome repair (GGR), and the second pathway is known as transcription-coupled repair (TCR), which is linked to dynamic transcription and is aimed at the transcribed strand of active genes.

Xeroderma pigmentosum (XP) is an inheritable genetic disorder where patients become extremely susceptible to ultraviolet exposure and have a predisposition to skin cancer. Based on complementation studies involving UV sensitivity of fused cells, XP was classified in 7 subgroups XP-A to XP-G. A UV-damaged DNA-binding component DDB (composed of subunits DDB1 and DDB2) is responsible for the inability of these cells to bind damaged DNA. DDB recognizes the DNA damage for GGR and recruits repair factors like XPC-HR23 [71, 72] to the sites of damage. In CS, patients are also hypersensitive to sunlight and show signs of premature ageing [73]. CS is caused by mutations in the CSA or CSB genes, and this leads to defects in TCR. The DDB2 and CSA proteins occur in large complexes (DDB-ligase complex) inside the cell that represents ubiquitin-ligase subunits CUL4A (cullin-4A), ROC1, and COP9 signalosome (CSN), which is the negative regulator of cullin-based ubiquitin ligases. Upon damage by UV, the DDB ligase complex translocates to the site of the DNA lesion resulting in its dissociation from the CSN following neddylation. The active DDB ligase complex then recruits XPC-HR23 to the damaged DNA. XPC, DDB2, and CUL4A are polyubiquitinated by the DDB ligase complex. Polyubiquitinated XPC binds DNA for repair activity whereas polyubiquitinated DDB is targeted for proteasomal degradation. The DDB ligase complex is thereafter displaced from the site of DNA damage.

## 6. Deubiquitinating Enzymes (DUBs)

Ubiquitination, like many other posttranslational modifications, is a dynamic and reversible process that depends on DUBs. The human genome encodes about 100 DUBs that are involved in processing of ubiquitin precursors and removal of ubiquitin or polyubiquitin from the target proteins [6]. DUBs are divided into five families based on their mechanism of action and phylogeny (reviewed in [74]). The first four families are papain-like cysteine proteases that include the ubiquitin-specific proteases (USPs), ubiquitin C-terminal hydrolases (UCHs), the ovarian tumor proteases (OTUs), and the Josephins. The fifth family is zinc-dependent metalloproteases. The DUBs are highly diverse in their structure and enzymatic activities and have a high degree of specificity for their substrates. The catalytic core domain of DUBs is responsible for the recognition and positioning of the enzyme on the ubiquitin substrates [75, 76]. In addition to the catalytic core domain, DUBs have ubiquitin-binding domains and protein-protein interaction domains that help in DUB function. According to available literature, there are seven DUBs that are involved in opposing the function of E3 ubiquitin ligases. As illustrated in previous sections, PCNA and FANCD2/FANCI ubiquitination is regulated by the DUB USP1 [6, 10]. USP1 seems to have functional importance in the PCNA-dependent postreplicative repair pathways as USP1 helps in relocalization of the translesion polymerase Pol*η* in UV-induced foci, and depletion of USP1 causes an increase in spontaneous and damage-induced mutagenesis [10]. However, in FA pathway, disruption of USP1 gene causes accumulation of the monoubiquitinated forms of FANCD2/FANCI but increases ICL hypersensitivity and genomic instability [49, 77]. Therefore, these activities of the DUBs ensure that the process of ubiquitination is a dynamic, reversible process that is optimally regulated in the cell.

## 7. The Ubiquitin-Proteasome Pathway as a Therapeutic Target

Ubiquitination has emerged as a key regulatory mechanism in DNA repair pathways and a powerful means for pharmacological intervention. Studies have shown how crucial DNA repair pathways are regulated by ubiquitin-dependent modifications. In addition, the TLS pathway is also regulated by ubiquitination and sumoylation. Thus, the different players including the E2 and E3 enzymes, the deubiquitinating enzymes (DUBs), and the proteins harboring the ubiquitin interacting motif (UIM) are all critical in recruitment and assembly of the repair complexes at the site of DNA damage. Therefore, in all likelihood, these posttranslational processes might provide attractive cancer therapeutic targets that inhibit the DNA repair pathways causing malignancies in the cell. The recent discovery that RNF8 plays a key role in ubiquitin-regulated double-strand break repair makes RNF8 an attractive target for new drugs. Thus, inhibitors to any ubiquitinated proteins that help in genome maintenance and repair are predicted to be potent antiproliferative agents. Discovery of such agents will open up new avenues for the treatment of cancer.

During times of cellular stress, the ubiquitin molecule, which is not produced in excess, could be limiting. In yeast and mammalian cells, the proteasome machinery is known to recycle the ubiquitin that is associated or conjugated to protein substrates that are condemned to be degraded. The deubiquitinating enzymes are capable of recycling the ubiquitin back to its monomeric form [78–80]. The proteasome inhibitor, bortezomib, can prevent both ubiquitin-mediated degradation and recycling of ubiquitin to monomeric forms thus causing a decrease in the monomeric ubiquitin pool [81]. Therefore, by depleting the pool of free ubiquitin, bortezomib can indirectly inhibit the DNA damage response and repair pathways, which could eventually lead to tumorigenesis.

Studies have shown that COP1, an E3-ubiquitin ligase, is overexpressed in human hepatocellular carcinoma (HCC). COP1 negatively modulates p53 by ubiquitination [82]. A recent report has shown how siRNA-mediated knockdown of COP1 could slow down growth and induce apoptosis in HCC cells [83]. Systemic suppression of COP1 in a xenotropic mouse model resulted in inhibition of HCC cell growth, thus making COP1 a therapeutic target in HCC. Much attention is being given lately to the cullin-regulated ligases (CRLs) which include ligases such as SCF complexes as these could be potential targets for chemotherapy. Recent reports have described small molecules that target the cullin-regulated E3 ligases [84]. Cullins are modified by the ubiquitin-like modifier NEDD8 for their function [81]. A specific inhibitor, MLN4924, of NEDD8 activating enzyme E1, had been developed, which inhibits the entire CRL subfamily [85, 86]. This small molecule inhibitor of NEDD8-E1 is presently being evaluated in several clinical trials. MLN4924 demonstrates significant antitumor activity *in vivo* in mice having human tumor xenografts at concentrations which are well tolerated. So, NEDD-activating enzyme inhibitors seem to have an enormous potential for treatment of cancer. The DUB family of enzymes could also hold promise for chemotherapy, as they have well-defined catalytic pockets, which make screening with small molecule inhibitors easy. Inhibitors of the ubiquitin carboxy-terminal hydrolase (UCH) family of DUBs have been reported with a decent amount of affinity and selectivity [87, 88]. Inhibitors of the SARS coronavirus DUB PLpro have been developed by screening a structurally diverse library of more than 50,000 compounds [89]. The discovery of this potent antivirus against SARS-CoV suggests that such drugs can be developed against the pathogenic DUBs such as SARS CoV DUB PLpro, without affecting the host DUBs.

## 8. Concluding Remarks

Ubiquitination has undoubtedly emerged as one of the major players of genome maintenance. The ubiquitin system not only senses the DNA damages but also repairs the damages in a highly regulated manner. The specificity of the E2-ubiquitin conjugating enzymes, the E3 ligases, and the DUBs with specific domains ensure that these activities are highly integrated and regulated. These posttranslational events are attractive targets for cancer therapy. There are still several mechanisms and questions that remain to be solved or answered. The targets of ubiquitination in the RNF8-regulated DSB pathway are still not clear. Similarly, several details of the FA pathway and the DNA damage bypass pathways are still being worked out. Targeting the USPs, SUMOylation, and neddylation pathways [90], as well as exploring the effects of deubiquination enzyme inhibitors [91], offers significant promise in the treatment of hematologic and solid malignancies.
